# The NIH Toolbox Cognitive Battery for intellectual disabilities: three preliminary studies and future directions

**DOI:** 10.1186/s11689-016-9167-4

**Published:** 2016-09-06

**Authors:** David Hessl, Stephanie M. Sansone, Elizabeth Berry-Kravis, Karen Riley, Keith F. Widaman, Leonard Abbeduto, Andrea Schneider, Jeanine Coleman, Dena Oaklander, Kelly C. Rhodes, Richard C. Gershon

**Affiliations:** 1 Translational Psychophysiology and Assessment Laboratory (T-PAL), MIND Institute, UC Davis Medical Center, Sacramento, CA USA; 2 Department of Psychiatry and Behavioral Sciences, University of California Davis Medical Center, Sacramento, CA USA; 3 Department of Pediatrics, University of California Davis Medical Center, Sacramento, CA USA; 4 Department of Pediatrics, Rush University Medical Center, Chicago, IL USA; 5 Department of Neurological Sciences, Rush University Medical Center, Chicago, IL USA; 6 Department of Biochemistry, Rush University Medical Center, Chicago, IL USA; 7 School of Medicine, Rush University Medical Center, Chicago, IL USA; 8 Morgridge College of Education, The University of Denver, Denver, CO USA; 9 Feinberg School of Medicine, Northwestern University, Chicago, IL USA; 10 Graduate School of Education, University of California Riverside, Riverside, CA USA

**Keywords:** Fragile X syndrome, Down syndrome, Assessment, Outcome measures, *FMR1* gene, Cognition

## Abstract

**Background:**

Recent advances in understanding molecular and synaptic mechanisms of intellectual disabilities (ID) in fragile X syndrome (FXS) and Down syndrome (DS) through animal models have led to targeted controlled trials with pharmacological agents designed to normalize these underlying mechanisms and improve clinical outcomes. However, several human clinical trials have failed to demonstrate efficacy of these targeted treatments to improve surrogate behavioral endpoints. Because the ultimate index of disease modification in these disorders is amelioration of ID, the validation of cognitive measures for tracking treatment response is essential. Here, we present preliminary research to validate the National Institutes of Health Toolbox Cognitive Battery (NIH-TCB) for ID.

**Methods:**

We completed three pilot studies of patients with FXS (total *n* = 63; mean age 19.3 ± 8.3 years, mean mental age 5.3 ± 1.6 years), DS (*n* = 47; mean age 16.1 ± 6.2, mean mental age 5.4 ± 2.0), and idiopathic ID (IID; *n* = 16; mean age 16.1 ± 5.0, mean mental age 6.6 ± 2.3) measuring processing speed, executive function, episodic memory, word/letter reading, receptive vocabulary, and working memory using the web-based NIH-TB-CB, addressing feasibility, test-retest reliability, construct validity, ecological validity, and syndrome differences and profiles.

**Results:**

Feasibility was good to excellent (≥80 % of participants with valid scores) for above mental age 4 years for all tests except list sorting (working memory). Test-retest stability was good to excellent, and convergent validity was similar to or better than results obtained from typically developing children in the normal sample for executive function and language measures. Examination of ecological validity revealed moderate to very strong correlations between the NIH-TCB composite and adaptive behavior and full-scale IQ measures. Syndrome/group comparisons demonstrated significant deficits for the FXS and DS groups relative to IID on attention and inhibitory control, a significant reading weakness for FXS, and a receptive vocabulary weakness for DS.

**Conclusions:**

The NIH-TCB has potential for assessing important dimensions of cognition in persons with ID, and several tests may be useful for tracking response to intervention. However, more extensive psychometric studies, evaluation of the NIH-TCB’s sensitivity to change, both developmentally and in the context of treatment, and perhaps establishing links to brain function in these populations, are required to determine the true utility of the battery as a set of outcome measures.

## Background

Although intellectual disability (ID) has been considered to be lifelong, with little promise for meaningful recovery of cognitive functions, recent advances in understanding of underlying genetic and neurobiological abnormalities of syndromic forms of several disorders, as well as progress in translation of targeted pharmacological and behavioral treatments, suggest that substantial cognitive gains may be possible and contribute to meaningful improvements in daily functioning and independence. Despite these exciting developments, there is little consensus on how cognitive improvements should be objectively measured. This paper presents preliminary research efforts to establish and validate the National Institutes of Health Toolbox Cognitive Battery (NIH-TCB) for individuals with ID, with a primary focus on its use as a series of cognitive endpoints for targeted pharmacological trials and other intervention studies.

An ID is a disability, originating before the age of 18, characterized by significant limitations in both intellectual functioning (typically IQ < 70) and adaptive behavior as expressed in conceptual, social, and/or practical skills (American Association of Intellectual and Developmental Disabilities; www.aaidd.org). ID is heavily represented in a wide range of neurodevelopmental disorders (NDD), including Down syndrome (DS), fetal alcohol syndrome, autism spectrum disorder (ASD), tuberous sclerosis, fragile X syndrome (FXS), Rett syndrome, and many other genetic conditions and syndromes with a spectrum of etiologies yet to be identified (idiopathic ID). In ASD, estimated to affect 1 in 88 children by the Centers for Disease Control (CDC), a meta-analysis of studies revealed a rate of ID as high as 75 % [1]; although more recent large-scale epidemiological studies reflect a rate of approximately 41 %.

Recent advances in understanding the molecular mechanisms underlying NDD through animal models [2–5] have led to targeted controlled trials with pharmacological agents designed to normalize molecular abnormalities, synaptic function, cognition, and behavior in humans with these conditions. FXS, the most common inherited form of ID, is by far the leading example of this translational effort. The disorder is caused by a mutation in a gene (fragile X mental retardation 1 or *FMR1*) on the long arm of the X chromosome, leading to the absence or reduction of its protein product, fragile X mental retardation 1 protein (FMRP). Lack of FMRP leads to abnormalities in dendritic structure and synaptic plasticity, as well as functional and structural brain abnormalities, and a cognitive phenotype characterized by deficits in executive function, including working memory [6, 7], inhibitory control [6–9], cognitive flexibility/perseveration [9], and selective and divided attention [9–11]; verbal short-term memory [10]; visuospatial memory [10]; processing of sequential and abstract information [12]; arithmetic reasoning [13–15]; and all domains of expressive and receptive language, most notably syntactic and pragmatic domains [16].

Extensive studies with two FXS animal models, the *fmr1* knockout mouse and *dfmr* Drosophila (fruit fly) mutant, have demonstrated abnormalities in metabotropic glutamate (mGluR) [3] and γ-aminobutyric acid (GABA) [17] receptor signaling due to loss of normal inhibitory control of dendritic translation in the absence of FMRP. In both models, numerous cognitive, behavioral, electrophysiologic, and morphologic (dendritic spine) phenotypes are normalized with mGluR5-negative modulators and GABA agonists. These discoveries paved the way for experimental treatment of the underlying neurobiology of the disorder in humans, including nine (to date) placebo-controlled, randomized trials of mGluR5-negative modulators (Roche—two phase 2a trials and a phase 2b trial; Novartis—a phase 2a [18] and two phase 2b trials [19]) and a GABA-B agonist (Arbaclofen, Seaside Therapeutics—a phase 2 trial [20] and two phase 3 trials in adults/adolescents and children [21]).

Despite substantial international efforts to evaluate the efficacy of these targeted treatments in adults and adolescents with FXS, the trials did not demonstrate significant clinical benefits based on the primary behavioral endpoints. Despite some promising positive indicators of improvement on the secondary outcome measures [20] or in post hoc analyses of potentially meaningful clinical subgroups [18], these trials were deemed “negative” or “failed” and none of these companies have been able to continue a FXS program. Given the overwhelming success with the animal models, the negative outcomes of human trials have been disheartening to families and have been a surprising and sobering call to investigators to better understand the limitations of the trials and to develop better designed studies with more sensitive outcome measures. Although ID is the hallmark feature of FXS, cognitive measures were not used to track treatment response, mainly due to a lack of consensus about which measures to choose, a lack of validation of existing measures in ID and/or FXS that could quantify short-term changes in cognition, and a desire to reduce distressing maladaptive behaviors which are typically of primary concern to caregivers [22–24].

DS is another neurodevelopmental disorder with promising therapeutic targets based on animal studies. DS is due to the presence of an extra copy of chromosome 21 (i.e., trisomy 21). The condition is relatively common, with an incidence of 1:1000 live births [25]. People with DS typically demonstrate deficits involving learning, memory, language, and movement. Most individuals with DS fall into the mild-to-moderate range of ID and have IQs comparable to males with FXS. The cognitive phenotype is characterized by deficits in verbal working memory and recall [26], cognitive flexibility [27–29], visual memory and learning [28, 30, 31], and planning and goal-directed problem-solving [32–34]. Deficits in the hippocampal and frontal systems involving memory and executive functioning are especially marked.

Development of a targeted treatment for individuals with DS has come from work with the Ts65Dn mouse model. The mice have segmental trisomy for a portion of mouse chromosome 16 that is orthologous to the long arm of human chromosome 21. Ts65Dn mice, like humans with DS, have profound deficits in memory and learning and demonstrate excessive inhibition (deficient long-term potentiation (LTP)) in the dentate gyrus. In the DS mouse model, cognitive deficits in object and declarative memory, as well as LTP deficits, are reversed with inverse agonists of alpha5 subunit-containing GABA_A_ receptors, leading to the use of these agents in human trials of DS [5, 35]. A phase 1b trial of RG1662 (Roche) for cognitive function in DS adults has been completed, and phase 2 trials in adults and children are in progress (clinicaltrials.gov; NCT02024789).

These early trials have generated an urgent need to develop and validate cognitive tools for tracking treatment response in ID for several reasons. First, parent ratings in the above-referenced FXS trials were affected by high placebo response rates (10–40 % improvement in baseline scores [19–21]) which substantially undermined the power to detect actual therapeutic benefits. In contrast, cognitive measures are a more direct assessment of performance and are less subject to placebo effects. Second, measures that are as close to the underlying neurobiological abnormalities of the disorder as possible are most desirable. Although it is possible to identify behaviors that are characteristic of the FXS phenotype, problematic behaviors are greatly affected by ongoing interaction with variations in the person’s environment (e.g., parenting, physical and social environment, responses to behaviors [36]). Cognition is likely to be affected by environmental factors as well but may be less prone to these confounds and better reflect true brain functional differences associated with neurobiology. Third, it is likely that some cognitive functions will respond to treatment more quickly than behavior or that the methods of cognitive assessment (error rate, response time) are more sensitive to positive changes in the brain’s capacity to attend to, process, and respond to information.

Many standardized cognitive measures (e.g., Wechsler Intelligence and Memory Scales, NEPSY-II, Leiter-R) appear to have face validity and utility for ID, but important limitations prevent them from being used as outcome measures in clinical trials. First, when these tests are given to individuals with ID, standardized scores (and often raw scores) are at or near the floor of the test range, severely limiting sensitivity [37, 38]. Second, many cognitive tests are developed to assess an individual’s abilities at a given time point for clinical assessment but are not suitable as clinical trial outcome measures due to lack of stability, practice effects, or lack of sensitivity to change during the treatment period. Third, lack of consensus on the best measures of cognition for persons with ID has led investigators to choose a wide variety of tests as outcome measures, often without knowledge of their psychometric properties (feasibility, reliability, validity) in persons with ID. This makes meaningful comparisons across studies and interventions, within and across disorders, difficult or impossible. Fourth, most measures do not demonstrate an association with real-life outcomes, such as adaptive behavior or functional skills. The validation of measures vis-à-vis functional outcomes is an important aspect in evaluating the results of clinical trials and labeling claims from the FDA perspective. Fifth, the broad range of severity of ID and high rate of behavioral and emotional disturbances make reliable and valid assessment difficult. Special and standardized procedures for handling these issues are likely to be essential in most studies but are rarely addressed, documented, or reported.

The NIH-TCB, a component of the NIH Toolbox for Assessment of Neurological and Behavioral Function, was developed to standardize evaluations in specific clinical populations for investigations of neurological development and change, disease recovery, and therapeutic interventions (www.nihtoolbox.org; [39, 40]). The NIH-TCB is a battery of extensively validated computer-administered cognitive tests with utility across childhood and adolescence, early adulthood, and old age. The NIH-TCB assessments were designed to minimize floor and ceiling effects which often are present in testing batteries designed for the general population. Therefore, there was good reason to believe that the assessments would be appropriate for individuals with ID. The initial validation study of 475 participants between 3 and 89 years, including 208 children, was completed several years ago [41]. The entire range of instruments, scoring criteria, and normative data from a randomly selected and stratified sample of 4500 individuals in the USA in this age range are now available. Recently, the NIH-TCB has been adapted for use on the iPad by the National Children’s Study to follow the development of children ages 3–21 years and their parents, and several of the subtests now have a developmental extension option to age 2. Despite its extensive development and validation in the general population, the NIH-TCB had not been evaluated for feasibility, reliability, validity, or sensitivity to change in individuals with ID.

Here, we report our experience with the NIH-TCB in three pilot studies of individuals with ID, focusing primarily on feasibility and identification of modifications that may be needed for ID populations (advantages and limitations for each test), as well as preliminary validity, reliability, and cross-syndrome comparisons. Three studies are presented together in sequential fashion in order to illustrate the progression, iterative process, and psychometric work involved in developing and validating cognitive outcome measures for atypical populations. The first study focused on feasibility in 31 patients with FXS utilizing earlier versions of two executive function tasks (flanker and dimensional change card sort, using keyboard responses) made available before the NIH-TCB was complete. The second study focused on 22 patients with FXS and 28 with DS, included all tests in the NIH-TCB, used Windows-based touch screen technology as employed in the standardization samples, and examined feasibility, test-retest reliability, and cross-syndrome comparisons. Finally, the third study, a pilot project within the first year of the multi-site funded grant “A Cognitive Test Battery for Intellectual Disabilities” (R01HD076189) progressed to use of the newly released tablet versions of the battery in 45 patients with ID (19 DS + ID, 10 FXS + ID, and 16 idiopathic ID (IID)), including examination of reliability and enhanced validity tests. Feasibility was further explored in developmental extensions of two NIH-TCB tasks and potential modifications of cross-validation measures.

## Materials and methods

### Participants

For study 1, to establish the initial feasibility of the NIH-TCB for individuals with ID, P. Zelazo shared two of the measures, flanker and dimensional change card sort (DCCS) [42] for pilot testing. These tests were administered to 31 patients with FXS [20 at UC Davis, 11 at Rush University Medical Center (RUMC); 27 males] between the ages of 5 and 36 years (mean = 19.3). Participants were recruited from fragile X clinics or from screening visits for clinical trials. The mean full-scale IQ (i.e., Stanford-Binet 5, Wechsler Scale, or Leiter-R) was 47.2 ± 16.1 and mean mental age was 5 years and 2 months with a range of 2 years and 1 month to 8 years and 0 months.

For study 2, participants included 22 patients with FXS (16 males; mean age 19.6 years, range 4.5–36.6) and 28 with DS (15 males; mean age 16.3 years, range 6.0–27.0). These individuals were all assessed at RUMC and recruited from the fragile X clinic or the Chicago area UPS for DownS family support organization. Mental age (measured from IQ testing or estimated by clinicians based on chart review, school records, and parent report of functioning) ranged from 2 to 10 years (mean 4.9 ± 2.0). Participants with FXS were on average 2 years lower (*p* < .001) on mental age than those with DS (5.9 vs. 3.7 years, respectively). In study 2, full-scale IQ (combined across available tests) ranged from 30 to 82 (mean 50.63 ± 13.7) and adaptive behavior (Vineland Adaptive Behavior Scales, Second Edition) composite scores ranged from 20 to 88 (mean 62.4 ± 19.8); however, these assessments were available on just 27 of the 50 participants.

For study 3, participants included 45 patients with ID [19 patients with DS, 10 patients with FXS, 16 patients with IID; 20 seen at UC Davis, 10 at University of Denver (DU), and 15 at RUMC], each with ID or borderline ID according to the Diagnostic and Statistical Manual of Mental Disorders, Fifth Edition (DSM-5) criteria (defined by a Stanford-Binet 5 full-scale IQ of <80) and impairments in adaptive behavior as measured by the Vineland Adaptive Behavior Scales. These participants were recruited from fragile X clinics at each site, DS and FXS support groups, research participant registries, special events for persons with developmental disabilities, and from notices distributed by family support foundations. In this study, all participants had a mental age of 3 years or higher, a chronological age between 6 and 25 years, no uncorrected vision or hearing impairments, no history of head trauma or other medical condition other than ID affecting cognition, and at least short-phrase speech with English as their primary language. Participants had a mean IQ of 51.3 ± 11.8, a mean chronological age of 15.8 ± 5.7 years, a mean mental age of 5.3 ± 2.0 years, and a adaptive behavior composite of 60.9 ± 17.0. The protocols for the studies received prior approval by the Institutional Review Boards at UC Davis, RUMC, and DU, and informed consent was obtained from each participant or their legal guardian.

### NIH-TCB measures

A thorough description of the NIH-TCB, including the rationale for test selection, neuroanatomical basis, and psychometric properties in a large representative sample of individuals from ages 3 to 89, are found in Weintraub et al. [39]. Additional details of the battery as specifically relevant to the pediatric population are found in Weintraub et al. [43].

#### Dimensional change card sort test 

DCCS [44] is a measure of cognitive flexibility. Two target pictures are presented that vary along two dimensions (i.e., shape and color). Participants are asked to match a series of bivalent test pictures (e.g., yellow balls and blue trucks) to the target pictures, first according to one dimension (e.g., color) and then, after a number of trials, according to the other dimension (e.g., shape). “Switch” trials are also employed, in which the participant must change the dimension being matched. For example, after four trials matching on shape, the participant is asked to match on color on the next trial and then switch back to matching by shape. Scoring is based on a combination of accuracy and reaction time (computed score, ranging from 0 to 10), and the test duration is about 4 min. Recent versions of this task include a developmental extension designed to extend the range of the assessment downward for those who find the original task difficult to understand. For example, participants are asked to match an image to one of two choices in which one is clearly similar and the other image is very different.

#### Flanker inhibitory control and attention test 

Flanker [44] is a measure of inhibition and visual attention. On each trial, a central directional target (fish for mental age younger than 8, arrows for ages 8 and older) is flanked by similar stimuli on the left and right. The participant chooses the direction of the central stimulus. On congruent trials, the flankers face the same direction as the target. On incongruent trials, they face the opposite direction. A scoring algorithm integrates accuracy, a suitable measure in early childhood/low mental ages, and reaction time, a measure more relevant to adult performance on this task, yielding computed scores from 0 to 10. There are 40 trials, and the test duration is about 4 min. This task also has a developmental extension. In the extension, participants begin by simply choosing the direction a single large fish is facing. The task becomes progressively more difficult by adding flanking fish of differing sizes and colors.

#### Picture sequence memory test (episodic memory)

Picture Sequence Memory [45] involves recalling increasingly lengthy series of illustrated objects and activities around different themes (e.g., “playing at the park,” “working on the farm”) that are presented in a particular order on the screen. For each trial, pictures appear in the center of the computer screen and then are moved one at a time into a fixed spatial order, as an audio file simultaneously describes the content of each (e.g., “Plant the tomatoes”), until the entire sequence is displayed on the screen. Then, the pictures return to the center of the screen in a random display and the participant moves them into the sequence that was shown. The score is derived from the cumulative number of adjacent pairs of pictures remembered correctly over 2–3 learning trials. Level of task difficulty is adjusted for the various age groups. Administration time is about 10 min. Theta scores are used for this test. Note that in study 2, the same form was used in test and retest, whereas in study 3, test-retest reliability of alternate forms A and B (different themes) was examined (randomized order). (In the normative studies, forms A and B were evaluated, and then scores adjusted for statistical equivalency using randomly equivalent cross-sectional administration. Score adjustments are applied depending on age group (e.g., ages 5–7, 8–59, 60+). No score adjustment was necessary for the 3–4 age group.)

#### List sorting working memory test 

List Sorting [46] requires immediate recall and sequencing of different visually and orally presented stimuli. Pictures of different foods and animals are displayed with accompanying audio recording and written text (e.g., “elephant”), and the participant is asked to state the items in size order from smallest to largest, first within a single dimension (either animals or foods) and then on two dimensions (first foods, then animals). The raw score is the number of items recalled and sequenced correctly, and the test duration is about 7 min.

#### Pattern comparison processing speed test 

Pattern Comparison [47] measures the speed of processing by asking participants to discern whether two side-by-side pictures are the same or not the same by touching “yes” or “no” (or a happy or frowning face for lower mental age). The raw score is the number of items correct in a 90 s period. The items are designed to be simple to distinguish. The test duration is about 3 min.

#### Oral reading recognition test 

For Oral Reading [48], the participant is asked to read and pronounce letters and words as accurately as possible. The items are administered by computer adaptive testing (CAT; continuously adapted depending on performance), and participant responses are scored by the examiner. For the youngest children, the initial items require identification of letters (as opposed to symbols) and identification of a specific letter within an array of four symbols. The test duration is about 3 min. A theta score is calculated for this test.

#### Picture vocabulary test

Picture Vocabulary [48] is a measure of receptive vocabulary administered in a CAT format. The participant is presented with an audio recording of a word and four photographs on the screen and is asked to select the picture that most closely matches the meaning of the word. This test duration is about 4 min. A theta score is used for this test.

Prior to the initiation of data collection, the research coordinator from each testing site was trained remotely by staff members from the NIH Toolbox Project at Northwestern University. The coordinators then trained all examiners at each site. Note that in studies 2 and 3, the starting points for each NIH-TCB test was based on each participant’s mental age rather than chronological age. All NIH-TCB tests have manualized instructions to ensure fidelity in administration and a set of practice items to aid participants in understanding and ensure compliance (see nihtoolbox.org). The website also includes technical manuals for each test covering validation, norming, and scoring algorithms.

### Concurrent validation measures (study 3)

See Table 1 for a list of NIH-TCB constructs and convergent validity measures chosen for study 3.

**Table 1:** Performance and parent-report observational (PRO) measures used for convergent validity by NIH-TCB construct in study 3

Construct	Toolbox task	Validation measures	Type
Cognitive flexibility	Dimensional charge card sort (DCCS)	KiTAP flexibility: errors and median Rxn time	Performance
		BRIEF-preschool flexibility scale BRIEF-school-age emotional control and shift scale	PRO
Inhibitory control and visual attention	Flanker	KiTAP go/no-go: errors, median, and SD Rxn time	Performance
		ABC hyperactivity subscale raw score	PRO
		BRIEF-preschool and school-age inhibit scale	PRO
		KiTAP distractibility: errors, median, and SD Rxn time	Performance
		SWAN attention subscale	PRO
Receptive vocabulary	Picture vocabulary	PPVT-4 raw score	Performance
Letter ID and word reading	Oral reading	WJ-4 letter/word ID raw score	Performance
Episodic memory	Picture sequence memory	Leiter-R forward memory raw score	Performance
		Leiter-R spatial memory picture score	Performance
Processing speed	Pattern comparison	KiTAP go/no-go: errors, median, and SD Rxn time	Performance
Working memory	List sorting	SB-5 verbal working memory	Performance
		BRIEF-preschool and school-age working memory scale	PRO

#### Kiddie Test of Attention Performance 

The Kiddie Test of Attention Performance (KiTAP; [49]) is an executive function battery comprised of eight subtests designed around an enchanted castle theme specifically designed to be accessible to young children. Based on our prior work on the feasibility, reliability, and validity of the KiTAP in FXS [49], we chose the flexibility, go/no-go, and distractibility subtests, which include reliable and validated scores matching well with several NIH-TCB constructs.

#### Peabody Picture Vocabulary Test, Fourth Edition 

The Peabody Picture Vocabulary Test, Fourth Edition (PPVT-4; [50]) is a norm-referenced test for measuring the receptive vocabulary. For each item, the examiner says a word, and the participant selects the picture that best captures the word’s meaning. This test was administered on a touch screen tablet and was chosen to cross-validate the picture vocabulary test of the NIH-TCB. The PPVT-4 also was used as a discriminant validity measure for several non-verbal NIH-TCB tasks, as it was in the normative studies [48].

#### Woodcock Johnson Tests of Achievement, Fourth Edition, Letter/Word Identification Test 

This is a measure of single word oral reading and letter identification [51]. This test was chosen to cross-validate the NIH-TCB oral reading test.

#### Leiter International Performance Test, Revised 

Spatial memory [52]: This is a measure of visuospatial memory. A matrix of child-friendly objects is displayed for 10 s, and then removed. The participant is asked to place the cards of the pictured objects in the correct locations on a blank matrix. Forward memory [52]: this is a measure of sequential memory span. The assessor shows the participant a grid with child-friendly pictures and taps the pictures in a specific order. The participant is then asked to tap the pictures in the same order.

#### Stanford-Binet Intelligence Scale, Fifth Edition—block span, verbal working memory index, and full-scale IQ 

For block span [53], the participant watches the examiner tap green cubes laid out in two rows on a page colored red or yellow. For easier items, the participant taps the blocks in the same sequence as the examiner, with increasing spatial spans. For more difficult items, requiring a higher working memory load, the participant observes the examiner tap the blocks as before but must tap all the blocks in one colored row in order first, followed by the blocks in the other colored row in order. The verbal working memory index  [53] has two types of test items, depending on difficulty: memory for sentences and last word. In memory for sentences, the participant repeats increasingly complex sentences said aloud by the examiner. In last word, the participant is read a series of from one to nine questions depending on level. First, the participant answers each question, and then, after all the questions, recalls and states the last word in each question.

For the Stanford-Binet IQ scores, we followed *z* deviation scoring methods described in detail in Sansone et al. [38], which provides significantly greater sensitivity and eliminates flooring in individuals in the low functioning range.

#### Caregiver report measures

The Aberrant Behavior Checklist (ABC) [54] is a symptom checklist for assessing problem behaviors of children and adults with intellectual disabilities. It includes scores on five subscales: irritability/agitation, lethargy/social withdrawal, stereotypic behavior, hyperactivity/noncompliance, and inappropriate speech. For this study, the ABC-community was used. The Behavior Rating Inventory of Executive Function (BRIEF) [55] is an 86-item parent or teacher rating to assess executive function and self-regulation in children and teenagers. Global executive composite is the combination of the behavioral regulation and metacognition indices. The strengths and weaknesses of attention-deficit/hyperactivity disorder symptoms and normal behavior scale (SWAN) [56] is an 18-item parent questionnaire assessing the symptoms of attention-deficit/hyperactivity disorder for children and adolescents.

**Fig. 1:**
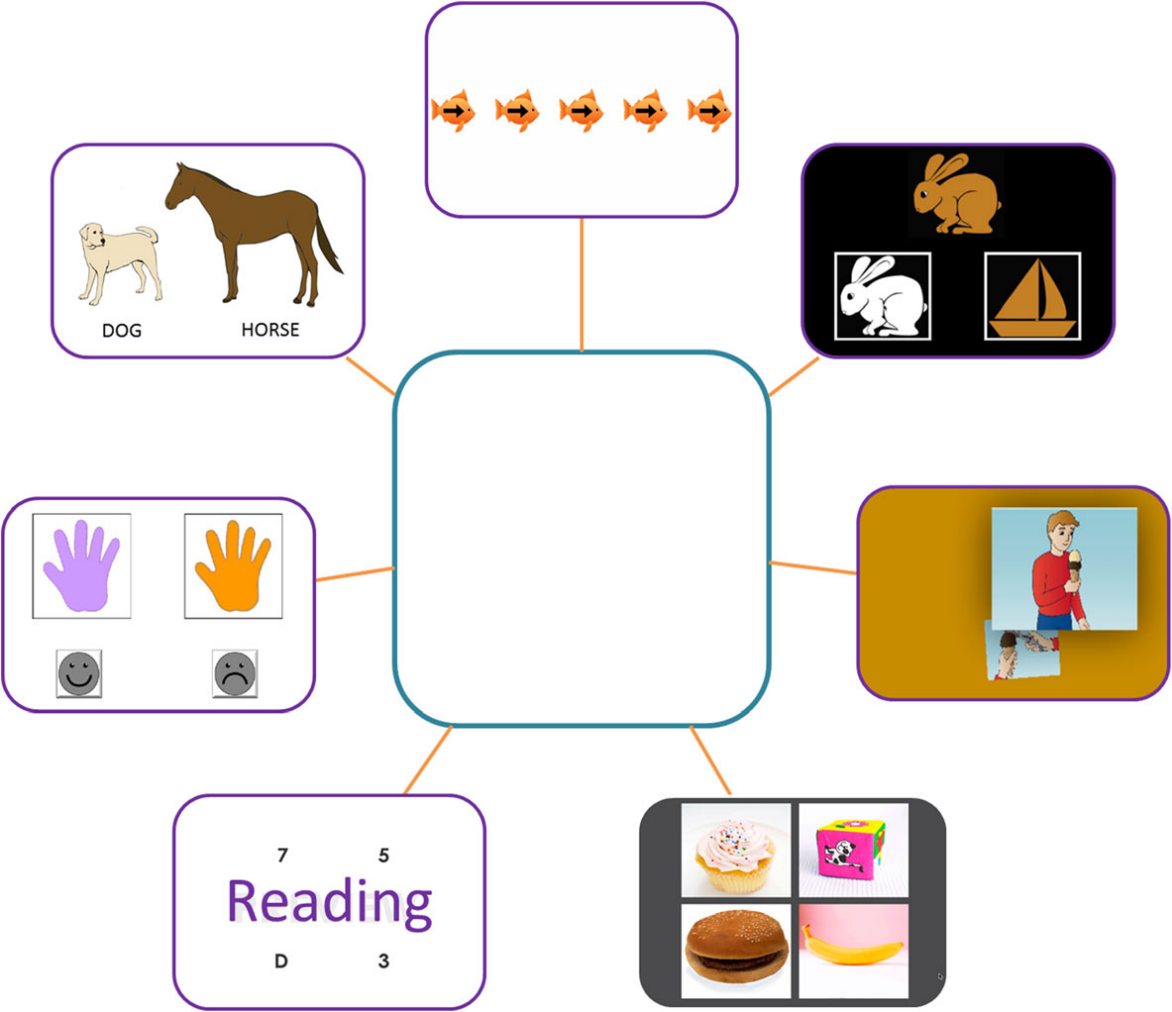
Story board/picture schedule used in study 3 to increase motivation and understanding of assessment visit schedule. The participant prize or a representation of the prize for compliance and effort is placed in the *center*, and with the examiner’s assistance, the participant checks off each completed task with a dry erase pen. NIH-TCB tasks from top (*clockwise*) depicted are flanker, dimensional change card sort, picture sequence memory, picture vocabulary, oral reading, pattern comparison, and list sorting

In each study, NIH-TCB measures were administered in a random order for each participant (DCCS and flanker only in study 1 and all seven measures in studies 2 and 3). In most cases, participants were cooperative and compliant during testing and responded to praise to maintain motivation and attention; however, in some cases, tangible rewards were used (e.g., a snack during a break or a small gift following completion of the battery). In studies 1 and 2, general notes were taken during test administrations to record behaviors or technical problems that might invalidate test results. These procedures were better standardized for study 3 and required examiners to check each test as valid or to record the reasons for invalid administration, which included when the participant (a) needed excessive prompting; (b) refused to be part of all of testing; (c) was unresponsive; (d) had poor task understanding (as judged by the ability to pass practice criteria and in some cases verbally explain what they were asked to do); (e) technical difficulties; (f) participant ill/emergency; and (g) others. These notes were later used to determine whether to exclude particular data points for examination of test feasibility and use in data analyses. For study 3, NIH-TCB and validation measures were administered across a 2-day period with frequent breaks to maximize compliance and minimize fatigue. A visual schedule/story board was used to increase structure, understanding, and predictability of the testing process and motivation in lower functioning participants (Fig. 1). The test-retest interval for study 2 was 13–95 days (mean = 37 days) and the interval for study 3 was 20–47 days (mean = 29 days). All data for study 3 was entered and managed within the Research Electronic Data Capture (REDCap) [57] system at UC Davis Clinical and Translational Science Center (CTSC).

### Data analyses

For analyses focused on test-retest reliability and convergent/discriminant validity, we used the raw, computed, and theta scores as described above for each test. In order to compare and contrast NIH-TCB profiles within and across diagnostic groups, we calculated age-corrected *z* scores representing the deviation of each participant’s performance on each test from the normative sample for his/her age (see [37, 38] for details of this scoring method). We also applied this transformation for the Stanford-Binet 5 IQ scores as previously described [38]. In both cases, the deviation scoring method is used to eliminate the flooring effects that are pervasive in the samples of individuals with ID when scaled scores are used. We calculated the toolbox crystallized composite (averaged from picture vocabulary and oral reading), toolbox fluid reasoning composite (averaged from DCCS, pattern comparison, list sorting, flanker, and picture sequence memory), and toolbox cognitive function composite (averaged crystallized and fluid reasoning scores) as described by Akshoomoff et al. [58]. We required 1 of 2 crystallized reasoning tests and 4 of 5 fluid reasoning tests to generate composite scores in these domains. Feasibility was calculated as the percentage of participants enrolled who completed each test yielding valid data (as determined by observation of testing by the examiner and the generation of a valid score). Test-retest reliability was estimated using intraclass correlation coefficients (ICC). Convergent and discriminant validity were examined using Pearson’s or Spearman’s correlations. NIH-TCB theta or raw scores were used to calculate test-retest reliability and to correlate with chronological and mental age and age-corrected deviation scores to correlate with age-adjusted IQ and adaptive behavior scores. Group comparisons of NIH-TCB *z* deviation scores were examined using ANOVA (collapsed across studies 2 and 3) followed by testing a priori contrasts based on the hypotheses of (a) greater deficits in executive function and working memory in FXS (compared to the other groups), (b) greater deficits in the language and episodic memory in DS (compared to the other groups), and (c) similar processing speed across groups. Given that the three groups were not equivalent on overall intelligence, we followed up with analyses using IQ as a covariate. In study 3, psychometric properties of the NIH-TCB in the ID samples (feasibility, reliability, and validity) were compared with those available from the pediatric sample (ages 3–16) from the general population used to develop the NIH-TCB [43].

## Results

### Study 1

All 31 patients with FXS in study 1 demonstrated the ability to use the touch screen for response, and the majority expressed positive effect and showed interest in use of this technology. A programming error resulted in two administrations of flanker that were not usable. For the remaining administrations, 24 of 29 (82.8 %) participants completed flanker and 83.8 % completed DCCS with valid scores as defined by Zelazo et al. [44] in general population studies (these rates were 89 and 90 % for those with a mental age of >3 years). The remaining participants either did not understand instructions adequate to reach practice criterion or needed too many prompts during testing to consider the results valid.

On flanker, participants made significantly more errors and demonstrated longer reaction times for incongruent than congruent trials (59 vs. 80 % correct, *p* = .003; 2363 vs. 1889 ms, *p* = .002). On DCCS, participants made more errors on trials involving a switch (change cognitive set) than without a switch (66.2 vs. 82.0 % correct; *p* < .001), although reaction times were not affected by trial type. These data demonstrated that the tasks produced expected effects on executive function in this sample. Flanker and DCCS scores demonstrated substantial range and variability, without flooring or ceiling, and showed modest Spearman’s correlations with full-scale IQ (flanker accuracy, rho = 0.65, *p* = 0.003; DCCS switch, rho = 0.52, *p* = 0.03), good to strong sensitivity to mental age (flanker accuracy, rho = 0.89, *p* < 0.001; DCCS switch, rho = 0.68, *p* = 0.001), but no association with chronological age (all *p* values >0.20).

### Study 2

#### Feasibility data

Of the 50 participants with FXS and DS seen in study 2, valid data were obtained from 66 % (list sorting) to 92 % (picture vocabulary) of the seven NIH-TCB tests (see Table 2). On review of the test administration notes, the two most common reasons for missing or invalid data were inattention and distractibility (e.g., responding in a valid manner and then losing focus and responding randomly) and lack of understanding of test instructions. There were a few administrations with technical problems that made it impossible for participants to maintain focus and motivation while problems were being fixed. A variable indexing the number of tests with successful data per participant (range 1–7) was created and correlated (Spearman’s rho) with chronological and mental age and with IQ and adaptive behavior composite scores (when available). Number of successful tests was significantly correlated with mental age (rho = .47, *p* = .001), IQ (rho = .63, *p* < .001, *n* = 27) and adaptive behavior (rho = .42, *p* = .030, *n* = 27).

**Table 2:** Study 2 feasibility, test-retest reliability, and examination of practice effects

	Feasibility (% of *n* = 50 valid)	Number (test-retest)	Visit 1, mean (SD)	Visit 2, mean (SD)	*t*	*p*	Cohen’s *d*	ICC
DCCS	84	17	3.32 (2.35)	3.89 (2.27)	−1.51	.15	.13	.88
Flanker	82	17	4.44 (1.98)	4.33 (2.33)	0.23	.82	.23	.75
List sorting	66	14	9.36 (4.31)	8.00 (4.47)	−1.56	.14	.31	.84
Oral reading	90	17	−3.94 (3.18)	−3.88 (3.31)	0.71	.49	.03	.99
Pattern comparison	90	16	29.53 (13.47)	35.35 (12.86)	2.08	.01	.44	.90
Picture sequence memory	78	17	−1.39 (0.72)	−1.12 (1.05)	1.45	.18	.46	.76
Picture vocabulary	92	17	−2.50 (2.13)	−3.05 (1.94)	−1.27	.22	.27	.77

Test-retest reliability measured by intraclass correlation (ICC). Practice effects measured by paired samples *t* tests. Effect size of difference measured by Cohen’s *d*

#### Test-retest reliability and examination of practice effects

Test-retest reliability coefficients and paired sample *t* tests comparing performance at baseline and retest are shown in Table 2. Test-retest reliability ranged from good (flanker, picture sequence memory, picture vocabulary) to very high (oral reading). Examination of differences between tests 1 and 2 showed significant practice effects for pattern comparison and potential practice effects for picture sequence memory (based on effect size).

#### Ecological validity

Although domain-specific validation tests were not available in study 2, examination of correlations with chronological and mental age were performed, as were correlations between NIH-TCB measures and adaptive behavior and full-scale IQ (18/25 IQ scores were derived from the Stanford-Binet 5 and 7/25 from the Leiter-R) for a subgroup of participants. As seen in Table 3, correlations with chronological age were weak. In contrast, mental age showed moderate to strong correlations with flanker, oral reading, and list sorting tests. Full-scale IQ (FSIQ) demonstrated consistent moderate to strong correlations with NIH-TCB tasks, and it correlated strongly with the NIH-TCB cognitive composite. Adaptive behavior was most strongly correlated with flanker, oral reading, and list sorting, and correlated well with the NIH-TCB composite.

**Table 3:** Study 2 ecological validity

	Chronological age	Mental age^a^	FSIQ^b^	Adaptive behavior composite
Dimensional change card sort	.23	.40**	.53**	.27
Flanker	−.02	.59***	.77***	.59**
Picture vocabulary	.32*	.22	.38	.26
Oral reading	.04	.61***	.71***	.73***
Picture sequence memory	.03	.14	.44*	.44*
Pattern comparison	.19	.24	.50**	.10
List sorting	.28	.54**	.61**	.50*
NIH-TCB cognitive composite	–	–	.75***	.61**

Data shown are Pearson’s correlations. *Note*: NIH-TCB age-adjusted *z* deviation scores were used to correlate with age-adjusted standardized measures (IQ, adaptive behavior), whereas computed or theta scores (unadjusted) were used for correlations with mental and chronological age

*p < .05; **p < .01; ***p < .001

^a^In study 2, mental age was either the actual mental age generated from individual IQ tests when test results were available or it was estimated by the examiner

^b^In study 2, IQ scores (Leiter-R or Stanford-Binet 5) were available from prior recent records in a subset of the sample (*n* = 25)

### Study 3

Descriptive statistics of gender, caregiver education, chronological and mental age, IQ, and adaptive behavior for the DS + ID, FXS + ID, and IID groups for study 3 are shown in Table 4. Primary caregiver education level was used as a proxy for socioeconomic status. In total, the participants in study 3 came from highly educated households with 65.9 % having at least a 4-year degree. The proportion of males in the FXS + ID and IID groups was almost twice what we observed in the DS + ID group. Based on parent/caregiver-report, ten individuals had a diagnosis of ASD, while three were reported to be of unknown status. Within the diagnostic groups, four individuals with FXS + ID and six individuals with IID were reported to have a diagnosis of ASD.

While the groups had comparable adaptive behavior composite scores (*F*(2,41) = 2.19, *p* = .13) and mean chronological ages (*F*(2,41) = .35, *p* = .70), they differed significantly by mental age (F(2,41) = 8.30, *p* = .001) and deviation IQ (*F*(2,42) = 9.69, *p* < .001). Post hoc comparisons using Tukey’s HSD test indicated that the average mental age and deviation IQ scores were significantly lower (*p* < .05) for the FXS + ID and DS + ID groups compared to the IID group. However, the FXS + ID and DS + ID groups did not significantly differ from one another (Table 4).

**Table 4:** Study 3 descriptive information by diagnostic group

	Total		DS + ID		FXS + ID		IID	
*n*	45		19		10		16	
Gender (% male)	71.1		47.4		90.0		87.5	
Primary caregiver education (% with at least a 4-year college degree)	65.9		68.4		50.0		73.3	
	*M*	SD	*M*	SD	*M*	SD	*M*	SD
Chronological age	15.80	5.69	15.01	6.32	16.81	5.73	16.13	5.04
Mental age equivalent (Stanford-Binet 5)	5.24	1.90	4.71	1.42	4.05	0.59	6.53	2.19
Full-scale deviation IQ score (Stanford-Binet 5; [38])	53.04	17.14	49.76	13.51	40.29	12.92	64.90	16.44
VABS-2 adaptive behavior composite	59.20	16.73	62.58	11.21	49.80	16.82	61.20	20.82

**Table 5:** Study 3 feasibility, convergent validity, and discriminant validity

	Feasibility (% valid)		Convergent validity		Discriminant validity	
	ID combined (study 3)	Age 3–15, general population^a^	ID combined (study 3)	Age 3–6, general population^a^	ID combined (study 3)	Age 3–6, general population^a^
DCCS	72.2 %	79.8 %	−.51*	.69***	.65***	.79***
			KiTAP flexibility errors	WPPSI-III block design	PPVT-4	
Flanker	77.8 %	83.7 %	−.61***	.60***	.61***	.67***
			KiTAP distractibility	WPPSI-III block design	PPVT-4	
Picture vocabulary^b^	100.0 %	83.7 %	.92***	.90***	.53***	.53***
			PPVT-4		Leiter-R forward + Spatial memory *z* score	BVMT-R + RAVLT
Oral reading^b^	94.4 %	98.1 %	91***	.96***	.56***	.53***
			WJ-4 letter/word ID	WRAT-IV	Leiter-R forward + Spatial memory *z* score	BVMT-R + RAVLT
Picture sequence memory	88.9 %	98.1 %	.57***	.50***	.64***	.58***
			Leiter-R forward + Spatial memory	NEPSY-II sentence repetition	PPVT-4	
Pattern comparison	66.7 %	94.4 %	−.40	.43***	.49**	.44***
			KiTAP go/no-go median RT	WPPSI-III processing speed	PPVT-4	
List sorting	52.8 %	95.7 %	.76***	.57***	.76***	.63***
			SB-5 verbal WM	NEPSY-II sentence	PPVT-4	

Validity data shown are Pearson’s correlations

*p < .05; **p < .01; ***p < .001

^a^Zelazo and Bauer [59]

^b^Estimates reported for the general population are aggregated across the entire child and adolescent sample ranging from 3 to 15 years old

#### Feasibility

The proportions of enrolled participants that were able to understand the NIH-TCB tasks and provide valid scores are shown in Table 5. For most of the tasks, these feasibility figures are comparable to children and adolescents from the general population, including DCCS, flanker, picture vocabulary, oral reading, and picture sequence memory. For list sorting, slightly more than half the sample was successful and yielded valid scores.

Although pattern comparison is a relatively simple task, for which we expected high feasibility figures based on study 2 (90 %), in this case, only two thirds of the participants were successful and yielded valid scores. Upon examination of the practice items and after reviewing participant response patterns, we noticed that practice items alternate between “same” and “different” correct responses. This pattern of alternating responses from participants often continued throughout the testing portion of the task, regardless of continued practice and examiner teaching, whether their responses were correct or not. Several individuals were either unable to break the rhythm of this pattern or began to misunderstand the task.

The number of valid tests completed (0–7) was significantly associated with mental age (rho = .70, *p* < .001) and deviation IQ (rho = .54, *p* < .001) but not with chronological age (rho = −.03, ns) or adaptive behavior (rho = .29, ns). The most commonly reported reasons from not obtaining valid data from participants in study 3 were difficulty passing practice items, excessive prompting during the test portion, refusal to respond, and questionable understanding. These issues were somewhat alleviated with the addition of the developmental extension levels for DCCS and flanker made available about half-way through study 3. Seven participants were administered the version in which developmental extension items are available. These seven individuals were between the ages of 8 years, 5 months and 23 years, 5 months (*M* = 13.98 ± 6.07). However, mental age estimates were between 3 years, 6 months and 6 years (*M* = 4.51 ± 0.89). Two individuals were diagnosed with FXS + ID, one with DS + ID, and the remaining four were diagnosed with idiopathic ID. Out of these seven, the developmental extension items were triggered for four participants on DCCS and three participants on flanker. Without the developmental extensions, these tests would not have been quantifiable. A breakdown of feasibility by mental age level was limited by small subgroup sample sizes in study 3, but by combining studies 2 and 3 (while acknowledging the limitations of mental age estimates in study 2), we had adequate numbers in each age bin (see Table 6). Feasibility was good to excellent (≥80 %) for above mental age of 4 years for all tests except list sorting. For above mental age of 5, more than three quarters of the participants provided valid data on all tests, and for above mental age of 7, feasibility was 100 % for the entire battery.

**Table 6:** Proportion of participants (%) by mental age group able to complete tests yielding scores judged by examiners and by data review to be valid (combined studies 2 and 3)

Mental age group	Number	DCCS	Flanker	Picture vocabulary	Oral reading	Picture sequence memory	Picture comparison	List sorting
3	26	65.4	76.9	88.5	88.5	61.5	69.2	34.6
4	20	85.0	80.0	90.0	90.0	85.0	90.0	50.0
5–6	25	84.0	88.0	92.0	92.0	96.0	84.0	76.0
7–8	12	100.0	100.0	100.0	100.0	100.0	100.0	100.0
9+	5	100.0	100.0	100.0	100.0	100.0	100.0	100.0

Note that in study 2, mental age was estimated based on chart review and examiner estimation. In study 3, mental age was measured by Stanford-Binet 5 IQ testing

##### Test-retest reliability and examination of practice effects

For study 3, the reliability statistics were similar or improved from study 2, with correlations in the mid .70 to high .90 (see Table 7). The exception was picture sequence memory with an ICC of .28. This likely reflected a lack of comparability between parallel forms A and B. In comparison, study 2 and the normative studies [59] used the same form A and achieved much higher correlations. (﻿As such, for group comparsions reported below, we only used data from form A). Aside from this difference, the test-retest reliability figures obtained in our sample with ID was comparable to those obtained from children and adolescents from the general population [59]. No significant differences in performance between test 1 and test 2 were observed for any of the NIH-TCB measures in study 3 (all *p* values >.10), although it should be emphasized that practice effects may be seen in larger samples.

**Table 7:** Study 3 test-retest reliability and examination of practice effects

	Number	Visit 1, mean (SD)	Visit 2, mean (SD)	*t*	*p*	Cohen’s *d*	Test-retest reliability, (21–49 days)	Test-retest reliability, general population, 3–15 years, (7–21 days)^b^
							ICC	ICC
Dimensional change card sort	27	3.52 (3.46)	4.29 (3.25)	−1.31	.20	.25	.74	.92
Flanker	32	4.59 (3.12)	4.38 (3.19)	.83	.41	.15	.94	.92
List sorting	26	7.50 (4.11)	8.30 (4.47)	-.85	.40	.36	.93	.86
Oral reading	34	901.21 (622.51)	916.91 (604.02)	-.29	.78	.05	.93	.97
Pattern comparison	20	28.65 (11.49)	30.08 (13.17)	−1.62	.12	.17	.86	.84
Picture sequence memory	30	394.84 (99.23)	404.15 (106.48)	-.38	.70	.07	.28^a^	.76
Picture vocabulary	36	914.08 (310.58)	885.14 (294.72)	1.16	.25	.19	.94	.81

Test-retest reliability measured by intraclass correlation (ICC). Practice effects measured by paired samples *t* tests. Effect size of difference measured by Cohen’s *d*

^a^Unlike study 2, alternate forms A and B were used at each visit (random order)

^b^Zelazo and Bauer [59]

##### Convergent and discriminant validity

Validity statistics of NIH-TCB measures in study 3 and comparison figures from the general population of children 3–6 years are shown in Table 5. Note that some validity measures chosen for each study differ; this should be taken into account when comparing correlations. In both samples, the language measures (oral reading and picture vocabulary) demonstrated excellent convergent validity (strong correlations above .90) and discriminant validity (correlations in the .50 range). The language convergent measures are very similar in the testing method and differ mainly in specific item content and time to administer (NIH-TCB tasks considerably shorter with CAT). The remaining NIH-TCB tasks showed adequate to good convergent validity with correlation values ranging from −.40 (pattern comparison raw score with KiTAP go/no-go median RT) to .76 (list sorting with SB-5 verbal working memory). In this sample with the chosen measures, discriminant validity was poor for the remaining NIH-TCB tests. With the exception of the language measures, the discriminant validity correlations were equivalent or higher than the convergent correlations. Limited discriminant validity of NIH-TCB tests was also reported for children from the general population normative study [59].

The parent-report convergent validity measures (BRIEF subscales, SWAN, ABC-C hyperactivity) did not correlate significantly with analogous NIH-TCB tests (listed in Table 1). The school-age BRIEF inhibition and the preschool BRIEF working memory correlated modestly with flanker and list sorting; however, the relatively small sample sizes for these correlations may have limited power to detect significant associations that may be present.

##### Ecological validity

**Fig. 2:**
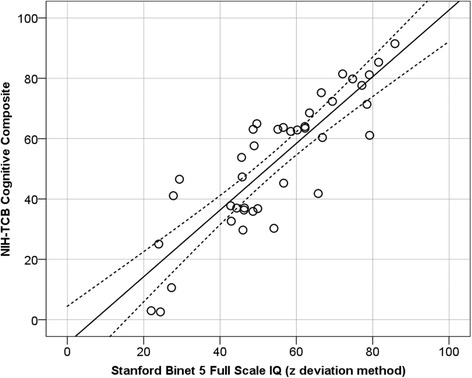
Scatterplot showing the association between the NIH-TCB cognitive composite and Stanford-Binet full-scale IQ (*z* deviation method). *Dotted lines* represent the 95 % confidence interval around the regression line. Note that the regression line and correlation (−12.76 + 1.17×; *R* ^2^ = .79) show that the composite is a strong predictor of IQ in these samples of individuals with ID

In the combined sample of individuals with FXS, DS, and IID, the NIH-TCB measures were strongly associated with mental age and deviation IQ (see Table 8). Indeed, the correlation between the NIH-TCB cognitive composite and the SB 5 full-scale deviation IQ was very strong (0.89; *p* < .001) and the regression line fell on the expected standard scores for each measure along most of the continuum of involvement (Fig. 2; e.g., SB-5 IQ of ~40 = NIH-TCB composite ~40). That is, in this sample, the NIH-TCB composite is a very strong predictor of FSIQ. Several NIH-TCB measures (DCCS, flanker, picture vocabulary, and oral reading) were modestly correlated with adaptive behavior, as was the NIH-TCB cognitive composite (*r* = 0.42, *p* < .05).

**Table 8:** Study 3 ecological validity

	Chronological age	Mental age equivalent	FSIQ (deviation)	Adaptive behavior composite
Dimensional change card sort	.31	.72***	.66***	.33
Flanker	.27	.61***	.70***	.36*
Picture vocabulary	.48**	.67***	.70***	.52**
Oral reading	.39*	.62***	.71***	.42**
Picture sequence memory	.34*	.55**	.57***	.16
Pattern comparison	.17	.45*	.46**	.20
List sorting	.57**	.49*	.52*	-.03
NIH-TCB cognitive composite	–	–	.89***	.42*

Data shown are Pearson’s correlations

NIH-TCB age-adjusted *z* deviation scores were used to correlate with age-adjusted standardized measures (IQ, adaptive behavior), whereas computed or theta scores (unadjusted) were used for correlations with mental and chronological age

*p < .05; **p < .01; ***p < .001

##### Group/syndrome NIH-TCB differences and profiles

**Fig. 3:**
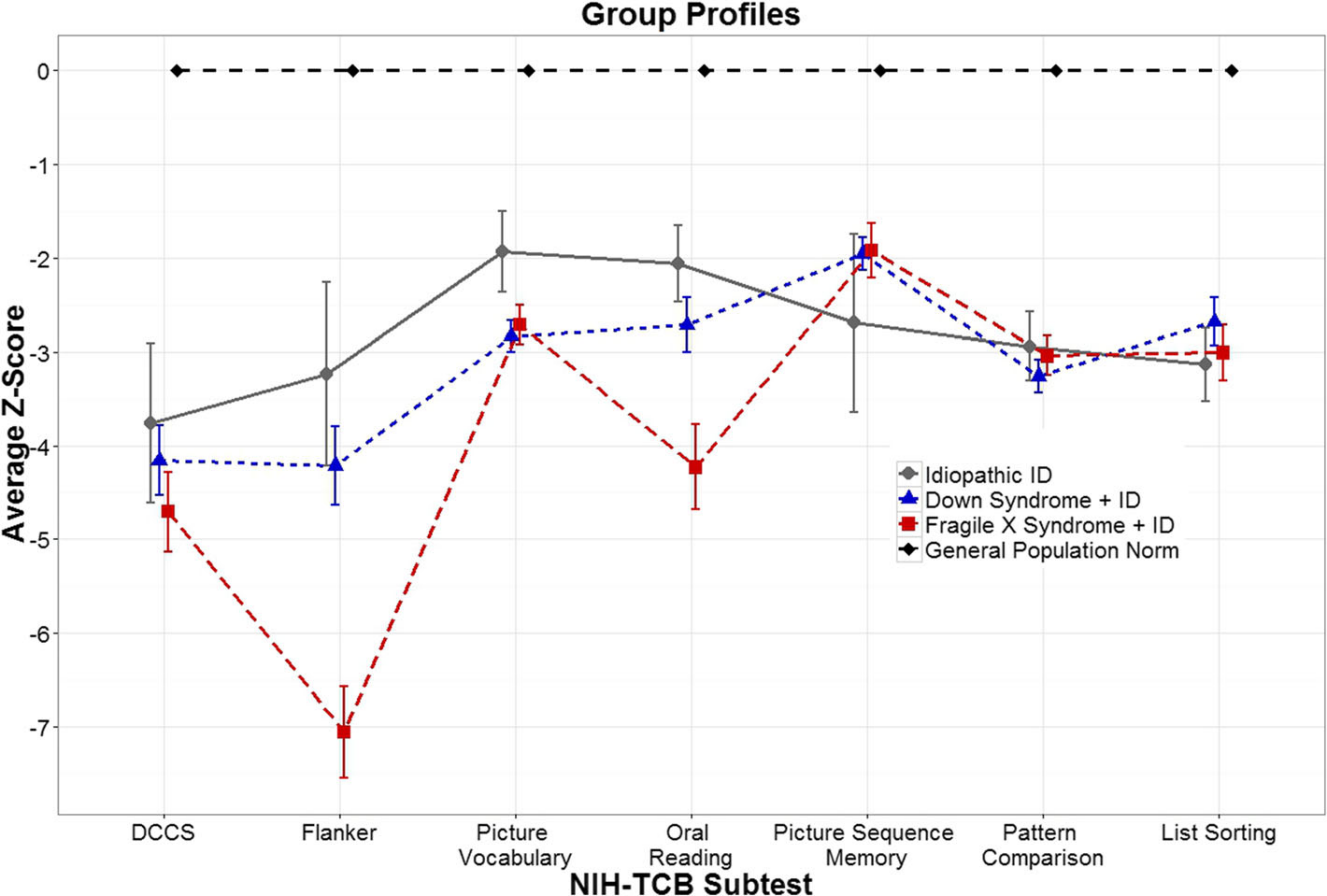
*Z* scores (±1 SEM) of each NIH-TCB subtest by group. *Z* scores (age-adjusted) reflect the number of standard deviations from the average (0 for all subtests) in the normative sample from the general population. For example, the FXS + ID sample had a mean performance on flanker that is greater than 7 standard deviations below average, adjusted for age. Note that for picture sequence memory, only data from form A is shown

Profiles of age-corrected NIH-TCB z deviation scores by diagnostic group (as well as the normative study or general population z scores of zero for comparison) are shown in Fig. 3. ANOVAs with group (DS + ID, FXS + ID, IID) as the independent variable and each of the NIH-TCB measures as the dependent variable yielded significant omnibus group effects for flanker [F(2,74) = 11.09, *p* < .001], picture vocabulary [F(2, 79) = 3.24, *p* = .04], and oral reading [F(2,80) = 7.16, *p* = .001].

Examination of planned contrasts for flanker demonstrated that FXS + ID and DS + ID combined had lower performance than IID [F(1,74) = 6.39, *p* = .01] and that FXS + ID performed significantly worse than DS + ID [F(1,74) = 15.79, *p* < .001]. The FXS + ID group mean for flanker was 7 standard deviations below general population norms. Furthermore, the effect of diagnostic group remained significant even when FSIQ was included as a covariate [F(2, 58) = 14.61, *p* <.001]. Additionally, when controlling for FSIQ, the differences observed between FXS + ID and DS + ID combined compared to the IID group [F(1, 58) = 10.41, *p* = .002], as well as those observed between the FXS + ID and DS + ID groups [F(1, 58) = 18.82, *p* < .001] remained significant. The a priori hypothesis of deficits on DCCS (cognitive flexibility) for DS + ID and FXS + ID, compared to IID, was not supported. Similarly, we hypothesized that individuals with DS + ID would show greater impairment in episodic memory as measured by the picture sequence memory task, however we found no significant group differences on this task [F(2, 57) = .95, *p* = .39]. 

The planned contrast for oral reading showed a significant deficit for FXS + ID relative to DS + ID and IID combined [F(1,80) = 13.17, *p* = .001]; however, a significant strength had been predicted. We also expected to find better reading performance for the IID group compared to DS+ID group, however our results did not support this hypothesis [F(1, 80) = 1.15, *p* = .29]. When FSIQ was included as a covariate the overall effect of diagnostic group on oral reading remained significant [F(2, 61) = 8.01, *p* < .001]. Additionally, when controlling for FSIQ, the DS + ID and IID groups together performed significantly better than the FXS + ID group [F(1, 61) = 13.28, *p* = .001], and the comparison of the IID and DS + ID groups remained non-significant [F(1, 61) = 2.73, *p* = .10] when controlling for FSIQ.

Picture vocabulary detected expected significant relative deficits for DS + ID compared to IID [F(1,79) = 6.28, *p* = .01], but the results did not support the hypothesis that FXS + ID would perform better than the other two groups [F(1,79) = .19, *p* = .67]. Also, the effect of diagnostic group on picture vocabulary did not remain significant when FSIQ was included a covariate [F(2, 61) = 2.34, *p* =.10]. As hypothesized, there were no significant group differences for processing speed [pattern comparison; F(2, 80) = .52, *p* = .60]. The highly variable profiles within groups and across tests relative to test norms (z = 0, SD = 1 for all tests, Fig. 3) suggest that the NIH-TCB has potential to detect group and syndromic cognitive profile differences.

## Discussion

In recent years, there has been an increasing pace of discovery of new genetic variants contributing to neurodevelopmental disorders, advances in the understanding of the neurobiology and phenotypes of these disorders, and a plethora of new treatments aiming to normalize brain neurobiology and improve functioning, with the potential to reverse cognitive deficits. The vast majority of clinical trials in FXS and many other disorders associated with ID have focused on social-emotional treatment targets including irritability/aggression, anxiety, and aberrant behavior more broadly. The research effort presented here to develop and validate cognitive outcome measures does not detract, or necessarily promote a shift in focus from, the critical goals of improving social, emotional, and behavioral functioning in patients with ID. Rather, it reflects an impetus to broaden the clinical target options available to investigators. In the three pilot studies presented here, we provide supporting evidence of the potential of the NIH Toolbox Cognitive Battery as outcome measures for ID, including preliminary results pertaining to feasibility, reliability, validity, and syndrome-related cognitive profiles.

Although several tests were feasible for a high proportion of participants (picture vocabulary, pattern comparison, oral reading), other measures in their original form (e.g., list sorting, picture sequence memory) were less often understandable especially for younger and/or lower functioning individuals. Given the desire for a battery applicable to a broad age range and developmental level, the developmental extensions of tests to a mental age of 2 years appear to increase feasibility. However, it is not yet clear whether the easiest items on these extensions measure their intended cognitive construct (for example, whether the easiest items of DCCS measure cognitive flexibility or simply the absence of this ability). Further, the tablet version of the NIH-TCB appears to have advantages for this population. For example, most participants are familiar with how to use and navigate a tablet from personal experience. Second, it allows more flexibility in testing positions (e.g., if the participant needs to move to a better location for testing). Third, the touch screen response, without the distraction of a keyboard or mouse, is much simpler and more intuitive. Fourth, working on the tablet appears more motivating to many participants. These interpretations are based mainly on our clinical observations. Although the feasibility figures for the tablet vs. touch screen versions of the battery are similar, we were stricter with determination of test administration validity in study 3, so any improved feasibility of the touch screen method was difficult to evaluate empirically. Future studies should be designed to more objectively determine the benefits of this mode of testing for individuals with ID. The preliminary data presented here suggest that the NIH-TCB feasibility for ID may increase substantially between mental ages of 3 and 5 years, although feasibility of the working memory test (list sorting) is more limited up to 5–6 years. A downward developmental extension of list sorting would be quite useful.

Test-retest stability across the period of likely treatment is a critical aspect of measure selection and impacts the ability to detect real change amid other factors leading to variability in measurement and impacts required sample sizes for clinical trials aiming to detect effects of particular magnitude. All of the NIH-TCB tasks demonstrated good to excellent test-retest reliability over an approximately a 4-week period. One exception to the strong test-retest stability of the battery was picture sequence memory (PSM) in study 3 (ICC = .28); however, in this study, two different forms (different story “themes”) were administered. Although statistical equivalency of the different forms was achieved in normative studies of individuals from the general population, test-retest reliability across forms was not reported. In study 2, use of the same PSM form/themes yielded good reliability and no clear evidence of a practice effect, although practice effects will require more careful examination in larger samples. Thus, our data provides evidence that the scores derived from the different forms of PSM are probably not equivalent, at least for individuals with ID. It may be inadvisable to use different forms of this test in a treatment study until equivalent scores and adequate test-retest reliability for this population are established. Also, the test-retest stability of the NIH-TCB tests substantially beyond 4 weeks cannot be determined by these results.

Construct validity, through examination of convergent validity correlations between NIH-TCB tasks and other measures purporting to tap the same cognitive constructs, and discriminant validity, aiming to show lower correlations with measures of different constructs, yielded mixed results in these samples. In study 3, we chose several measures from the KiTAP to examine convergent validity with NIH-TCB executive function tasks, based on our prior studies showing its feasibility, reliability, and validity [49]. However, unlike in our prior work with older patients enrolled in clinical trials, in this pilot study of younger, perhaps somewhat lower functioning individuals, we obtained fewer useable data from this test. For the data that were obtained, convergent validity estimates were very similar to those obtained in the normative sample of children ages 3–6 years [59], suggesting no major falloff in validity for individuals with ID in these chronological and mental age ranges. Convergent validity was very good for picture vocabulary and oral reading, owing in part to the nearly identical mode of assessment on the NIH-TCB and validity measures (PPVT-4 and Woodcock-Johnson word reading). For many tasks, similar to the data obtained from the normative sample of 3–6-year-old children, there was little evidence of discriminant validity. This is likely due to the lack of divergence of cognitive domains of function in this mental age range, and the fact that receptive vocabulary (chosen as the discriminant validity measure for the non-verbal tests) is highly correlated with *g* (overall IQ) which is in turn well correlated with all NIH-TCB tests. In future studies, it appears essential to utilize alternative measures that are less strongly associated with *g* to examine discriminant validity.

The ability of a measure to detect expected cognitive phenotypes and differences is another aspect of test validity. As executive dysfunction is an extensively documented and prominent aspect of the FXS phenotype [6–11], the NIH-TCB was successful in measuring substantial inhibitory control and attention deficits among these participants. Similarly, although the battery has limited language measures, the picture vocabulary test did yield lower scores for participants with DS, a group who has previously been found to score lower on standardized tests of vocabulary than typically developing mental age matches [60–64] and individuals with other forms of ID [62]. Future studies with much larger sample sizes, and including participants with ID of different etiologies, will further establish the utility of the NIH-TCB and extend its construct validity.

There are several important limitations of this research. Many of the participants, especially those with FXS and DS, were patients seen in clinics or were previously screened and enrolled in clinical trials. While these individuals may represent the population of individuals to be studied in clinical research, they may be more affected than the larger populations of these syndromes. The preliminary studies reported here have focused primarily on feasibility and validity in relatively small samples; more rigorous work examining the psychometrics of the NIH-TCB measures in larger samples of individuals with ID are needed before they can be given a “green light” for use as primary outcomes in clinical trials or other applications. Information on the sensitivity of these measures to actual changes in cognitive and daily functioning is needed. Also, the lack of discriminant validity for some of the measures warrants further work to determine whether this reflects a weakness in measurement specificity or a developmentally appropriate lack of differentiation of cognitive skills. Additional work by other investigators is needed to replicate and extend these findings and methodological concepts and to compare the utility and performance of the NIH-TCB in ID to other existing measures or other batteries in development. Along these lines, collaboration is strongly encouraged, so that the methods are well standardized and data are comparable across studies, laboratories, and clinical populations. This will promote future multi-site clinical research, which is increasingly essential for maximizing statistical power to address research questions about rare NDDs and to generalize results across cultural and ethnic groups.

The results of the pilot studies were used as a supportive evidence for a larger-scale project now underway (“A Cognitive Test Battery for Intellectual Disabilities”; R01HD076189). The NIH-TCB for ID is being further refined and adapted and will be formally validated utilizing much larger samples (*n* = 150 per group) of individuals with FXS, DS, and other forms of ID (idiopathic or various etiologies). Assuming the aims of the project can be achieved, namely, to show that the NIH-TCB tests are feasible, reliable, valid, and sensitive to change in individuals with ID, there are a number of research directions and questions that can be pursued. The most obvious application would be to move the tests into clinical trials as outcome measures to track cognitive changes associated with treatment. This will be the best way to examine whether the battery, or components of it, is sensitive to pharmacological or behavioral intervention. This will take time and numerous studies to evaluate its performance in this capacity. Also, some of the NIH-TCB tasks may be more suitable for use as outcome measures in clinical trials than others. For example, the fluid reasoning measures, such as flanker, DCCS, and pattern comparison (all of which have a timed component), and perhaps list sorting and picture sequence memory, may be more likely to change over a relatively short treatment period than crystallized measures of oral reading and picture vocabulary, which reflect acquired knowledge. Second, the battery may be quite useful in developmental/longitudinal studies of cognitive changes in individuals with ID. Third, given that several of the tests have established links to brain functions (working memory/frontal [65, 66]; inhibitory control/frontostriatal [7]; episodic memory/hippocampus [67]; processing speed/white matter development [68]), the battery may aid in understanding the neuropsychological basis of cognitive impairments in specific syndromic forms of ID, which may in turn lead to more targeted cognitive interventions. For example, prior work on the neuropsychological aspects of FXS and DS, especially deficits in executive functions [9, 10], have led to trials of computer-based working memory training (see [69, 70]; “Cognitive Training for Fragile X Syndrome”, clinicaltrials.gov). The NIH-TCB executive function tasks might be employed as outcome measures in these types of cognitive training studies.

Translational research programs in FXS, DS, and other ID conditions continue to benefit from animal models which have provided paradigms elucidating neural mechanisms underlying cognitive and behavioral abnormalities. However, as has been seen in the recent FXS trials, translation from mouse studies to demonstration of human therapeutic benefits has been extremely challenging. We suspect that phenotypes of *Fmr1* knockout mice may differ phenomenologically and mechanistically from that of human patients. For example, “anxiety” or “memory” as measured by the elevated plus and Morris water mazes, respectively, may differ substantially from mechanisms and contextual expression of such problems in humans with FXS. A critical goal for the translational efforts in ID going forward is to establish measures across species that share the same or similar neurobiological mechanisms. In this way, candidate drugs can be compared across species with more confidence that they are acting on the same pathways. In this regard, it is noteworthy that cognitive tasks developed for mice, including measures of attention, inhibitory control, working and object memory, and pattern discrimination using touch screen technology [71–74], are available and could in the future be compared across species with some of the NIH-TCB tasks. This should facilitate translation of findings from animal to human studies and will maximize the potential for the discovery of truly disease-modifying interventions.

## Conclusions

The three preliminary studies reported here provide the first psychometric support for the utility of the NIH Toolbox Cognitive Battery for individuals with ID, a population that has historically posed significant challenges for clinicians and investigators to develop feasible and valid cognitive outcome measures. With forthcoming larger-scale validation, this battery has potential to facilitate the detection of cognitive changes associated with pharmacological and behavioral interventions with web-based technology that is scalable and reliable across multi-site studies.

## Abbreviations

ABC-C: Aberrant Behavior Checklist-Community
ANOVA: Analysis of Variance
ASD: Autism Spectrum Disorder
BRIEF: Behavior Rating of Executive Function
CAT: Computer Adaptive Testing
CDC: Centers for Disease Control and Prevention
CTSC: Clinical and Translational Science Center
DCCS: Dimensional Change Card Sort
DS: Down Syndrome
DSM-5: Diagnostic and Statistical Manual of Mental Disorders, Fifth Edition
DU: University of Denver
FDA: Federal Drug Administration
FMR1: Fragile X Mental Retardation 1
FMRP: Fragile X Mental Retardation 1 Protein
FSIQ: Full-Scale IQ
FXS: Fragile X Syndrome
GABA: γ-Aminobutyric Acid
ID: Intellectual Disability
IID: Idiopathic Intellectual Disability
IQ: Intelligence Quotient
KiTAP: Kiddie Test of Attentional Performance
LTP: Long-Term Potentiation
mGluR: Metabotropic Glutamate Receptor
MIND: Medical Investigation of Neurodevelopmental Disorders
NDD: Neurodevelopmental Disorders
NIH: National Institutes of Health
PPVT-4: Peabody Picture Vocabulary Test, Fourth Edition
RT: Reaction Time
RUMC: Rush University Medical Center
SWAN: Strengths and Weaknesses of Attention-Deficit/Hyperactivity Disorder Symptoms and Normal Behavior
TCB: Toolbox Cognitive Battery
VABS-2: Vineland Adaptive Behavior Scale, Second Edition
WJ-III: Woodcock-Johnson, Third Edition
